# Thrombospondins and remodeling of the tumor microenvironment

**DOI:** 10.20517/2574-1209.2018.40

**Published:** 2018-10-10

**Authors:** Olga Stenina-Adognravi, Santoshi Muppala, Jasmine Gajeton

**Affiliations:** 1 Department of Molecular Cardiology, Cleveland Clinic, Cleveland, 44195, USA.

**Keywords:** Thrombospondin, angiogenesis, inflammation, cancer

## Abstract

Vascular remodeling defines cancer growth and aggressiveness. Although cancer cells produce pro-angiogenic signals, the fate of angiogenesis critically depends on the cancer microenvironment. Composition of the extracellular matrix (ECM) and tumor inflammation determine whether a cancer will remain dormant, will be recognized by the immune system and eliminated, or whether the tumor will develop and lead to the spread and metastasis of cancer cells. Thrombospondins (TSPs), a family of ECM proteins that has long been associated with the regulation of angiogenesis and cancer, regulate multiple physiological processes that determine cancer growth and spreading, from angiogenesis to inflammation, metabolic changes, and properties of ECM. Here, we sought to review publications that describe various functions of TSPs that link these proteins to regulation of cancer growth by modulating multiple physiological and pathological events that prevent or support tumor development. In addition to its direct effects on angiogenesis, TSPs have important roles in regulation of inflammation, immunity, ECM properties and composition, and glucose and insulin metabolism. Furthermore, TSPs have distinct roles as regulators of remodeling in tissues and tumors, such that the pathways activated by a single TSP can interact and influence each other. The complex nature of TSP interactions and functions, including their different cell- and tissue-specific effects, may lead to confusing results and controversial conclusions when taken out of the context of interdisciplinary and holistic approaches. However, studies of TSP functions and roles in different systems of the organism offer an integrative view of tumor remodeling and a potential for finding therapeutic targets that would modulate multiple complementary processes associated with cancer growth.

## INTRODUCTION

The studies of cancer initiation and progression have focused on the molecular and cellular signalling events that lead to changes in cancer cell differentiation, proliferation, and apoptosis, all of which initiate uncontrolled growth and spreading. The roles of immunity and of the response of the whole organism to cancer cells have been historically appreciated, but the role of the microenvironment in the initiation, progression, and spreading of cancer has become a more active field of study only recently. It has been accepted that cancer cells are constantly forming in a healthy body^[1,2]^ but they do not survive. They may remain dormant for years due to the healthy microenvironment, which does not support the tumor growth. The tumor microenvironment, which prevents or promotes cancer growth, consists of stromal and vascular cells, immune and inflammatory cells. Additionally, the extracellular matrix (ECM) and secreted signals that these cells generate promote or restrict cancer cell division and migration^[3,4]^. The progression of cancer depends on the complex interplay between the tumor cells and the tumor microenvironment. Targeting the components of the tumor microenvironment is now a recognized powerful tool of cancer therapy and prevention of spreading and recurrence.

The development of tumor vasculature depends on angiogenesis, accompanied by inflammation, which is an important factor in predicting tumor vascularization, growth, and spreading. Targeting the tumor vasculature has been an active approach in finding new therapeutic targets for many years^[5]^ but, unfortunately, has not fulfilled the expectations of cancer therapies due to significant side effects and adverse events in tumors in response to hypoxia ^[6]^. It has become clear that we have a limited appreciation of pathological processes associated with the tumor microenvironment and, as a result, inadequate understanding of potential therapies that may improve the microenvironment and restrict tumor growth.

Tumor angiogenesis and the recruitment of immune and inflammatory cells into the tumor rely on the composition of ECM ^[7]^. One important event that occurs during tumor progression is the stiffening of the ECM, caused by the deposition of collagen and fibronectin, leading to increased proliferation and tumor advancement^[8]^. Cancer-associated fibroblasts are important contributors of ECM stiffening^[9]^. Tumor ECM is also modified by vascular and blood cells that release proteases and chemoattractants and deposit ECM to promote angiogenesis, additional recruitment of vascular and inflammatory cells, and inflammation^[10]^.

Tumor inflammation is closely associated with the tumor aggressiveness and metastasis^[11]^. Activated cancer and vascular cells produce chemoattractants and pro-inflammatory signals to recruit inflammatory cells from blood. The accumulation of inflammatory cells in a tumor is an important prognostic index that has been successfully used to evaluate the aggressiveness of cancer in conjunction with other indexes that describe the proliferation rate of cancer cells and their migratory potential. CD68, a marker of macrophages is one of the 16 markers evaluated in Oncotype DX, a clinical test that is used to make therapeutic decisions and predict the aggressiveness of breast cancer^[12,13]^. The recruitment and retention of inflammatory cells in tumors depends on the ECM composition^[14]^.

This article reviews the contribution of thrombospondins (TSPs), a family of secreted ECM proteins, in regulation of the cancer microenvironment and the initiation and progression of tumor growth that is defined by the vascular and ECM remodeling and inflammatory response. It is becoming clear that TSPs affect every pathological process associated with cancer advancement and are key protein regulators of the tissue remodeling that occurs with cancer growth.

## TSPS AND CANCER

The ECM is complex and ever-changing. All the cells in a tumor constantly remodel the ECM and deposit growth factors, proteases, pro-inflammatory and chemoattractant proteins into ECM. The composition of ECM defines whether it will support cancer growth, angiogenesis, and inflammation by providing specific growth- and migration-promoting signals and changing the physical properties of the tissue. ECM contains structural components, e.g., collagens, and secreted proteins that define the interactions of the cells with the structural elements of the ECM. One of the influential groups of proteins that regulates the interactions between structural proteins and cells are TSPs. This family of proteins consists of five members in humans (TSP-1 - TSP-5). The five members of the family share a high level of homology and a number of properties^[15]^. However, they have unique domains and protein sequences that render each of the five TSPs distinct in their interactions with the ligands in ECM and with cell surface receptors. As a result, TSPs have distinct functions in tissue remodeling and regulation of cancer growth^[16]^. Furthermore, a strong and repeated association of TSP expression or suppression in various cancers highlights their role in cancer regulation^[17]^.

## TSP-3, TSP-4, AND TSP-5

TSPs of group B (TSP-3, TSP-4, and TSP-5) are evolutionarily older proteins with fewer domains than in the group A TSP proteins^[18]^. Group B TSPs are important in embryonic development^[16,19]^ and participate in the activation of embryonic tissue remodeling programs^[20]^. TSP-3 and TSP-5 are involved in the regulation of growth plate organization and limb length^[21]^. Complete deletion of TSP-3 and TSP-5 leads to reduced limb length, which signifies their prominent role in skeletal growth ^[21]^. Not surprisingly, TSP-3 is also linked to cancer angiogenesis, metastasis and invasion in osteosarcoma patients^[22]^.

TSP-4 is one of the highly upregulated genes (in the top 1%) in several types of cancer, e.g., gastric cancer ^[23–25]^ and breast cancer^[26–28]^. Its expression is upregulated in stromal tissue of invasive breast and gastric adenoma cancers^[20,29]^. A recent study suggests that the loss of miR-142, resulting in high expression of TSP-4, enhances hepatocellular carcinoma (HCC) invasion and progression. Therefore, targeting TSP-4 may be an important strategy to treat HCC^[30]^. Increased expression of TSP-4 in ECM promotes invasion of the breast cancer cells^[20]^. Another study stated that TSP-4 mRNA expression in fibroblasts was stimulated by cancer cells, suggesting that TSP-4 is an important novel marker in the detection of diffuse-type gastric adenocarcinomas^[29]^. Flexible heteroarotinoid compounds coordinate growth, apoptosis and differentiation of cancer cells. One of the compounds of this group, SHetA2, inhibits angiogenic effects by decreasing the secretion of TSP-4, along with vascular endothelial growth factor A and fibroblast growth factor, in ovarian and renal cancers^[31]^.

TSP-4 promotes cancer angiogenesis and growth in mouse models of breast cancer^[32]^. Knocking out TSP-4 in mice resulted in smaller tumors with decreased numbers of endothelial cells and lower levels of angiogenesis markers. Conversely, a P387 variant of TSP-4 that is a more active variant of TSP-4 in cellular effects and interactions with ligands^[32]^, had increased cancer angiogenesis and tumor growth^[32]^. Although the vascular cells appear to be the main source of TSP-4 in breast cancers^[32,33]^, *in vivo*, the cancer cells themselves also produce small amounts of TSP-4 that appear to be sufficient to support angiogenesis and cancer growth even in TSP-4 deficient animals ^[32,33]^. Complete deletion of TSP-4, in both the host and the cancer cells, is required in order to document effects on tumor growth^[33]^. In addition to these effects in tumors, TSP-4 promotes adhesion and migration of leukocytes^[34]^. Thus, TSP-4 is a pro-angiogenic^[32]^ and a pro-inflammatory protein^[35]^ that supports tumor growth by activating multiple complementary pathways.

TSP-4 mediates the effects of transforming growth factor beta (TGF-β), a master regulator of ECM and inflammation, on angiogenesis^[33]^. The direct roles of TSP-4 in ECM regulation remain poorly understood. However, it is clear that TSP-4 regulates collagen production and can prevent fibrosis in tissues^[36]^.

On the other hand, TSP-4 serves as a tumor suppressor in colorectal cancer and suppresses *in vitro* tumor colony formation^[37]^. Epigenetic profiling studies revealed that hypermethylation of the TSP-4 promoter leads to its inactivation and loss of TSP-4 tumor suppressor function in cutaneous T cell lymphoma^[38]^. The opposite effects of TSPs on cancer cells and on the cancer microenvironment are a recurrent theme when studying the roles of TSPs in cancer regulation, leading to controversial findings that are difficult to explain. Ultimately, the results from *in vivo* studies, where TSPs levels have been manipulated, should be considered to help define physiological roles for individual TSPs in cancer regulation.

## TSP-1 AND TSP-2

The more recently developed group of TSPs, which includes TSP-1 and TSP-2 and is termed “group A”, appeared in evolution along with the development of the cardiovascular system^[19,39]^. These two proteins have several C-terminal domains homologous to group B proteins but differ from group B TSPs in their N-terminal protein parts^[40]^. The newly acquired in evolution domains of TSP have important roles in regulating vascular tissue remodeling: they harbor sequences associated with the anti-angiogenic action of these two proteins^[41,42]^ and with the inhibition of metalloproteinases (MMP)^[43,44]^. One of the N-terminal domains in TSP-1 contains a sequence that binds TGF-β: TSP-1 is a major activator of latent TGF-β^[45–47]^.

The anti-angiogenic properties of TSP-1 and TSP-2 have been studied for over 30 years^[48]^. TSP-1 and TSP-2 inhibit endothelial cell proliferation, migration, and apoptosis^[49–52]^. The decreased expression of TSP-1 and TSP-2 in tumors and surrounding tissues has been reported^[53–55]^, and animal studies confirmed their anti-angiogenic and tumor-preventing action^[56]^. Furthermore, TSP-1 expression has been associated with cancer dormancy^[57,58]^; merely suppressing or overexpressing TSP-1 is enough to reverse the patterns of tumor growth in specific anatomical areas with differential expression of TSP-1^[59–63]^.

In addition to the direct effects of TSP-1 and TSP-2 on endothelial cells, the regulation of angiogenesis may be an indirect consequence of regulation of MMP activity^[44]^, binding of growth factors and regulation of their availability and activity^[64]^, and regulation of functions of the immune and inflammatory cells^[65,66]^.

The effects of TSP-1 on cancer cells are sometimes inconsistent with its anti-angiogenic and anti-cancer effects observed in *in vivo* studies. TSP-1 has adhesive properties that support cancer cell growth^[67–69]^, promote metastatic properties of breast cancer cells^[70]^, facilitate the invasion of squamous cell carcinoma^[71]^, breast cancer cells^[72]^ and melanoma^[73]^, may increase proliferation of cancer cells^[74]^, and decrease cancer cell apoptosis^[75]^.

## INTEGRATIVE APPROACH TO UNDERSTANDING TSP ROLES IN CANCER

These contradictory effects of TSPs on cultured cancer cells and on the fate of a tumor *in vivo* have not been explained. To better understand their significance, these contradictory effects should be considered in a context of complex relationships between the cancer cells and the entire organism: prevention of a tumor growth not only relies on the cancer cell properties alone but also requires a concerted response of the body that involves the activation of immune responses, the recognition of cancer cells, and clearance of these cells. Tumor development occurs only when multiple body systems fail to eliminate cancer cells from the system. Cancer cells are constantly forming in different tissues and also circulate in blood^[76]^ but fail to attach and initiate a tumor growth when the microenvironment (including ECM of tissues), regulation of angiogenesis, and responses of the immune system are normal^[77,78]^. Dysregulation of metabolic, immune, and tissue remodeling processes is what leads a single cancer cell to progress to a tumor rather than being recognized, killed and eliminated. The physiological balance of cancer cell attachment, proliferation and mobility versus their recognition, apoptosis, and elimination define the fate of each cancer cell that forms in the body.

The *in vivo* effects of TSP-1 and TSP-2, suggest that these proteins activate a whole -organism anti-angiogenic and anti-cancer program that ultimately leads to a decrease in cancer growth or to cancer cell dormancy^[58]^. Thus, the positive effects of TSP-1 and TSP-2 on cancer cell proliferation could be considered as a part of this program, which initiates anti-cancer defense in multiple body systems. For example, increased proliferation of cancer cells due to TSP-1 signaling may render the cancer cells more susceptible to the elimination by natural killer cells^[79]^. Furthermore, TSP-1 signaling may facilitate activation of p53, a regulator of apoptosis^[80,81]^. Promoting cancer cell proliferation and invasion may result in better responses from T-cells due to expression of cancer-specific antigens and their circulation in blood. Similar to the therapeutic approaches designed by humans, e.g., chemotherapy and radiation treatment of cancers, the natural body responses may be the most efficient when the cancer cells are rapidly growing. Understanding why TSPs have cell-specific responses and seemingly contradictory effects would explain how they protect from cancers in the case of TSP-1 or promote cancer growth in the case of TSP-4. Better understanding these complex and sometimes contradictory properties of TSPs will only be possible by developing an integrative approach and more holistic view of the pathological and physiological processes regulated by these proteins, considering the fact that they affect multiple organ systems.

Newly developed integrative approaches to cancer therapies have pushed the field to better understand the causes of cancer and the mechanisms, by which tumors grow and spread. As a result, inflammation and the metabolic changes have become the focus of many studies that investigate how the cancer microenvironment is regulated^[82,83]^. A growing body of evidence connects increased levels of blood glucose and insulin and chronic inflammation with cancer initiation and progression^[84,85]^. While the association of diabetes and cancer has been known for many years^[86–89]^, recent studies suggested that even post-prandial elevations in blood glucose and/or insulin increases the risk of cancer. The glycemic load (GL, a measure of the increase in post-prandial blood glucose caused by food) and/or the high dietary glycemic index (GI, another index that estimates the effect of foods on post-prandial blood glucose) were associated with a risk of breast cancer^[90–94]^; with lung cancer^[95]^; prostate cancer^[93,96]^, especially with its aggressive form^[97]^; endometrial cancer ^[93,98]^; ovarian cancer^[93]^; and digestive tract cancers (esophageal, stomach, colorectal, liver, gallbladder, and pancreatic)^[93,96,99–103]^. The emerging evidence stresses the importance of diets low in GI and GL and reduction of carbohydrates in diets as a part of healthy nutrition and lifestyle to prevent cancer development and recurrence^[104–106]^. The connection between chronic inflammation and cancer has been known for a long time: e.g., an association between the hepatitis and the liver cancer has been well recognized and studied^[107,108]^, the existence of cancers caused by pancreatitis and Crohn’s disease has been known and accepted^[109,110]^, and the connection between the infection with italicize and stomach cancer has been confirmed^[111,112]^. Diabetes, pre-diabetes, and metabolic syndrome are associated with chronic inflammation^[113–116]^ and can be induced by the chronic inflammation in growing adipose tissue^[117–119]^ and pancreas^[120–122]^. Thus, metabolic dysfunction appears to increase the risk of cancer directly (due to an increased blood glucose and insulin) and by increasing the inflammation. TSP-1, that normally restrains angiogenesis and prevents the growth of a tumor, is downregulated by high blood glucose levels in many tissues^[123,124]^, thus, providing a link between the elevated blood glucose and cancer. TSP-1 has been shown to be downregulated by a microRNA, miR-467, in response to hyperglycemia^[124]^. Inhibition of miR-467 using an antagonist effectively inhibited hyperglycemia-induced breast cancer growth in mice^[125]^. Furthermore, decreased levels of TSP-1 are associated with higher inflammation in tissues, probably due to its ability to stimulate phagocytosis in macrophages and to promote the resolution of inflammation^[66,126]^. Therefore, increasing the levels of TSP-1 may stop or prevent the growth of tumors in multiple complementary ways by decreasing cancer angiogenesis and promoting the resolution of cancer inflammation.

## ANTI-CANCER TSP-BASED APPROACHES

The functions of TSP-2, TSP-3, and TSP-4 in regulation of cancer growth are not well enough understood to identify potential therapeutic approaches based on the regulation of expression of these proteins or on their specific ligands and cell surface receptors. However, TSP-1, a TSP family member discovered and purified from platelets^[127]^ 40 years ago, has been a target for developing strategies to modulate its levels or to take advantage of its interactions with ligands in ECM and on the cell surface.

Multiple attempts to use TSP-1 fragments to inhibit cancer growth have been described in the literature. Adenovirus-mediated gene therapy containing an antiangiogenic fragment of TSP-1 inhibited the growth of the human leukemia xenograft in mice^[128]^. Gene therapy with a fragment of TSP-1 inhibited the growth of human breast carcinoma, MDA-MB-435, *in vivo* in mice^[129]^. The delivery of the fragment together with p53 resulted in a synergistic effect and decreased the cancer growth more than the TSP-1 fragment or p53 administered separately. Linear and cyclic peptide TSP-1 mimetics have been tested in anti-angiogenesis therapies^[130–135]^.

The interaction of TSP-1 with CD47 was shown to mediate multiple effects of TSP-1^[136]^. Targeting this interaction, with the goal of increasing angiogenesis, led to an unexpected outcome - angiogenesis inhibition^[137]^. The peptide, designed to block the interaction of TSP-1 with CD47, named TAX2, increased the binding of TSP-1 to CD36 and disrupted vascular endothelial growth factor receptor 2 activation and subsequent downstream NO signaling. This peptide was also tested in experimental animal cancer models and inhibited angiogenesis and growth of melanoma^[137]^, pancreatic carcinoma^[137]^ and neuroblastoma^[138]^. It was also effective in preventing the spread of melanoma^[139]^. The 4N1 peptide, based on the sequence of TSP-1 domain that binds CD47, was successfully used in a mouse model as an anti-leukemia agent^[140]^. The interaction of TSP-1 with CD47 was found to be important in multiple processes related to tumor growth. For example, blocking the signaling through CD47 conferred protection of normal tissue to irradiation through activation of autophagy pathways^[141,142]^. Modulation of the anti-tumor immunity by CD47 in T cells by this pathway has been described^[143]^. Thus, this TSP-1-CD47 interaction appears to be a valuable therapeutic target.

One of the cell-specific effects of TSP-1, mediated by its interaction with CD47, limits cell survival in response to radiation^[144]^, suggesting that antagonizing this interaction would provide a selective radioprotection for normal cells and tissues. Another tissue- and cell-specific approach targeted a miRNA regulating TSP-1 production: miR-467 increases in a cell- and tissue-specific manner in response to hyperglycemia and silences the production of TSP-1^[124]^. Thus, antagonizing this miRNA slows down the growth of certain cancers without affecting TSP-1 production in response to high glucose in other tissues^[125]^.

Some unexpected outcomes from using anti-TSP-1 strategies highlight the complexity of TSP-1 interactions and its functions. The domains involved in regulating angiogenesis, TGF-β activation, and MMP inhibition are localized in N-terminal part of TSP-1, while interaction with CD47 depends on the C-terminal domain of the protein. However, based on the results of peptide studies, the domains are functionally associated, such that blocking the interaction with one receptor also changes the interactions of distant domains with other receptors and ligands^[64,145]^. Due to the multiple cell-specific functions, the effects of TSPs on various cells types that are involved in tumor progression should be also taken in the account when considering pharmacological interventions that target the expressions of TSPs or block their interactions with their ligands.

ECM proteins appear to be good targets for therapy because of their extracellular localization and relatively easy availability for drugs. However, very few ECM proteins have become successful therapeutic targets. Most ECM proteins, including TSPs, have a complex multi-domain structure with a number of ligands on the ECM and cell surface. The combined effect of TSP interactions with other ligands and receptors may not only depend on their protein levels in tissues but also on the availability of ligands and receptors on the cell surface. Ultimately, the systemic effects caused by inhibiting TSPs or regulating their production should be considered. Successful strategies need to be based on tissue- and cell-specific evidence such that interactions do not alter the functions or expression of TSPs elsewhere.

**Figure 1.**
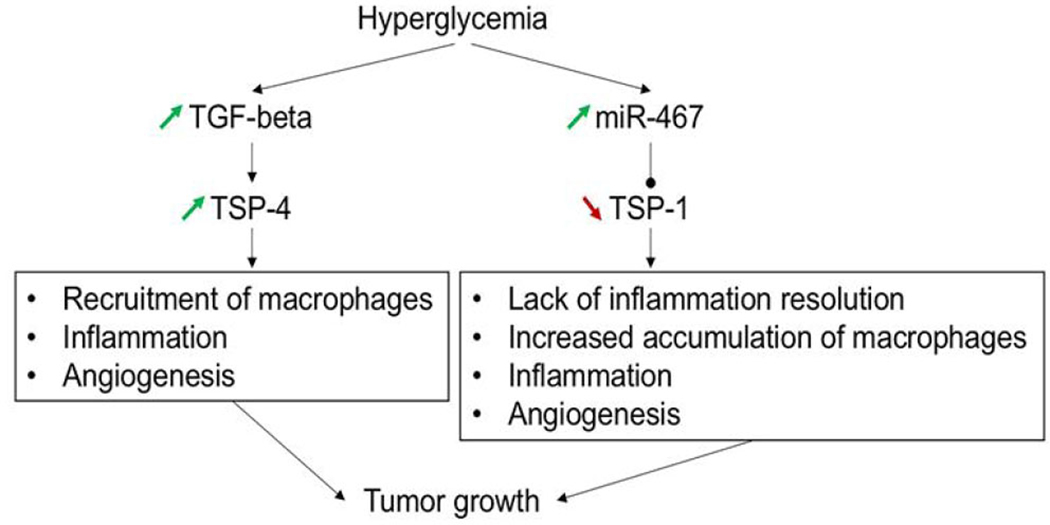
Hyperglycemia promotes cancer growth by regulating thrombospondin (TSP)-1- and TSP-4-dependent pathways. Upregulation of TGF-β in response to hyperglycemia leads to upregulation of TSP-4. TSP-4 is a pro-angiogenic protein that also promotes recruitment of macrophages and other leukocytes into tissues and increases local inflammation. Upregulation of miR-467 in a tissue-specific manner blocks TSP-1 production. In the absence of the anti-angiogenic pressure of TSP-1, cancer angiogenesis is increased. In the absence of TSP-1, the resolution of inflammation is impaired. Increased inflammation and angiogenesis promote cancer growth in the absence of TSP-1. TSP-1 and TSP-4 pathways converge and complement each other to promote the tumor growth

Interactions between TSP pathways further complicate the final outcomes. For example, studies of the effects of hyperglycemia on breast cancer suggest that TSP-1-dependent pathways may synergize with TSP-4-dependent pathways. Higher expression of miR-467 in response to high glucose was associated with inhibition of TSP-1 production^[123,124,146]^ [Figure 1]. In addition to its anti-angiogenic effects, TSP-1 is a regulator of inflammation and functions of macrophages^[147]^. TSP-1 is known to regulate the production of cytokines by macrophages^[148–150]^, to stimulate micropinocytosis^[151]^, motility^[152]^, to activate toll-like receptor 4 pathway in macrophages^[153]^, and to promote the resolution of inflammation^[150,154]^. Hyperglycemia changes the levels of multiple ECM proteins, including the master ECM regulator TGF-β^[155,156]^. Higher levels of TGF-β have been detected in the cancers of diabetic patients^[157,158]^, and blocking TGF-β signaling leads to better outcomes in animal models^[159–161]^. It was reported recently that increased levels of TGF-β led to increased production of TSP-4. Unlike TSP-1, TSP-4 is pro-angiogenic^[162,163]^ and increases accumulation of macrophages and other leukocytes in tissues *via* increased recruitment into tissues^[34,35]^. Increased levels of TSP-4 combined with decreased levels of TSP-1 would promote a pro-angiogenic and inflammatory microenvironment leading to tumor growth [Figure 1].

**Figure 2.**
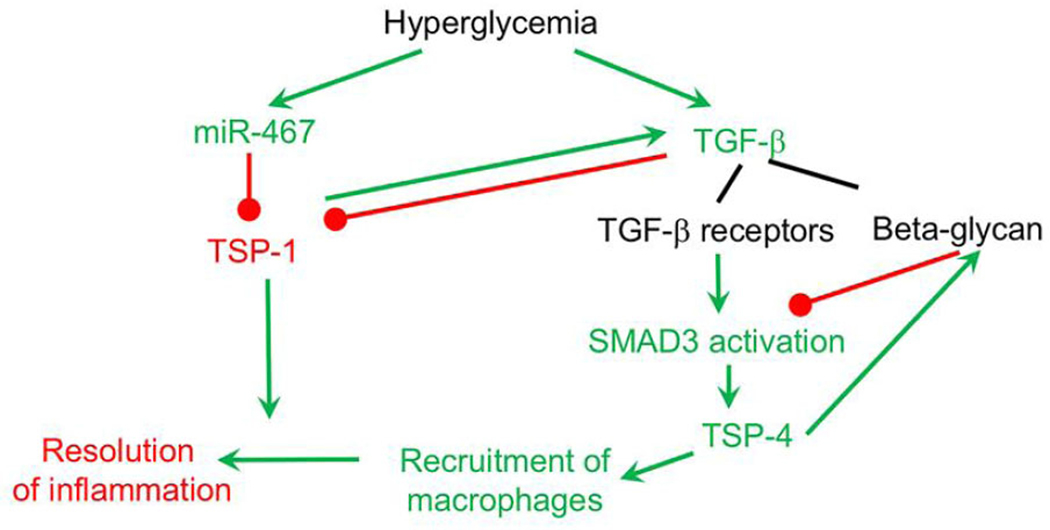
Interaction of hyperglycemia-regulated thrombospondin (TSP) pathways. TSP-4 increases recruitment of macrophages, while TSP-1 is needed for the resolution of inflammation. In response to hyperglycemia, TSP-1 levels are downregulated by increased levels of miR-467. TSP-4 is upregulated as a result of upregulation of TGF-β and activation of SMAD3. Upregulation of TSP-4 result in increased recruitment of macrophages into the tumor. In the absence of TSP-1 and resolution of inflammation, the accumulation of macrophages increases. In a feedback loop, TSP-4 increases the levels of an inhibitory TGF-β receptor beta-glycan. TGF-β further decreases the production of TSP-1. In a feedback loop, TSP-1 is an activator of TGF-β. Green arrow and text = upregulation in response to hyperglycemia; red arrow and text = downregulation in response to hyperglycemia. TGF-β: transforming growth factor beta

In addition to functional interaction, TSP pathways interact at the mechanistic level. For example, TSP-1 activates TGF-β^[45,47,164]^ and is downregulated by TGF-β^[163]^ but TSP-4 is a mediator of the TGF-β effects^[163]^ and, in turn, modulates the expression of one of the TGF-β receptors, beta-glycan^[165]^, thus, controlling TGF-β signaling [Figure 2].

## CONCLUSION

TSPs become available to many cell types after they are secreted and incorporated into ECM. They have multiple interactions and functions, which depend on the availability of specific cell surface receptors on each cell type at any given moment. The final outcome of modulating TSP levels is determined by a combined effect from their actions in multiple cell types and organs, from the tumor itself to the immune system and vasculature.

While multiple targets may potentiate the effects of modulation of TSP expression and functions, the complexity of TSP interactions requires an unbiased testing of the effects of potential anti-cancer therapies in *in vivo* models. When the interactions and mechanisms are dissected and understood, TSPs may become desirable targets for the integrative anti-cancer approaches.
